# Scalma

**Published:** 1887-10

**Authors:** Rush Shippen Huidekoper

**Affiliations:** Dean Veterinary Department University of Pennsylvania


### Art. XXX.—SCALMA.

BY RUSH SHIPPEN HUIDEKOPER, M.D.

*Dean Veterinary Department University of Pennsylvania.*

The differentiation of the various diseases which have popularly been included under the terms of distemper and influenza until comparatively recent date, has been so slow and so tardily accepted by the majority of practitioners, that we have been subjected to constantly see announced in the daily papers the appearance of some new disease. These new diseases of the populace and of the empiric are to us but the epidemic outbreak, or the more severely manifested form of some ordinary contagious disease. We treat several cases of different troubles in the same stable, without having the time or seeing the necessity of explaining them to the owner, when suddenly one of them spreads to the rest of the stable in an epidemic form, and our clients will not understand that all of the animals have not suffered from the same illness.

There is, however, one of the contagious fevers of the horse, which has constantly been confounded with other diseases, and which has not been separated from them in our English text books. As this disease has received no proper name in English I shall use for it the name given by Professor Dieckerhoff, of Berlin, who first described it in the Adams Wochenschrift XXIX. in 1885.

*Etymology.*—The term scalma is derived from the old German word *Scalmo*, *Scelmo*, *Schelm*, which indicates roughishness or knavishness, as great nervous irritability, especially of the temper, is one of the characteristic, almost diagnostic, symptoms of this disease. The term "Heimtûckische Krankheit," signifying malicious, treacherous or mischievous, is also employed in German for the same trouble. I am not aware of any name in English or French which has been applied to it.

As I am opposed to employing in veterinary medicine any of the nomenclature of human medicine, except for identical, simple and inflammatory diseases, or for intercommunicable contagious diseases, I will not offer the term whooping cough as a name, but will suggest a certain similarity between the latter disease in man and scalma in the horse.

*Definition.*—Scalma is a contagious and infectious febrile disease of the horse, with local lesions of the bronchi, trachea and larynx, which is evidenced by cough. It is further characterized by great irritability of temper. It occurs as a stable plague—that is, in enzootic form with, however, great variations in the susceptibility of the animals to contract it. It is rarely fatal except from complications.

*Incubation.*—The period of incubation is from six to seven days, but the disease may develop in two days after exposure or it may delay its appearance for ten days. It spreads through a stable slowly, developing at times in a horse placed in a stall where the previously sick one had stood, or it may pass next to an animal several stalls away. One attack is usually protective.

*Symptoms.*—The symptoms are ushered in by fever, in which the acceleration of the pulse and respiration is in no way in accord with the great elevation of temperature. With the appearance of the fever is developed a diffuse bronchitis which is, however, *subacute*, both in its character and in its course. At times the bronchitis may extend to the trachea, larynx, pharynx, or even to the nasal fossæ.

In two or three days a trifling grayish, albuminous discharge occurs, which continues, variable in quantity, for eight to fourteen days, or even may last for three weeks. The cough is short, rough and painful, spasmodic in its occurrence and in character. The slight watery or slimy discharge may become more profuse, purulent, or even "rusty," if the bronchitis has extended to the neighboring structures. Pharyngeal discharge may take place.

The respiration is moderate and only affected during an access of cough, or in complicated cases. The pulse undergoes but little quickening. The temperature rises rapidly to 39° C., 40° C., and in some cases even to 41.5° C. (107½° F.) The latter temperature usually, but not always, indicates complication by pleurisy. In ordinary cases the temperature drops in two or three days after the appearance of the cough. The hide is dry and rough with the hairs on end.

Emaciation may be rapid. The mucous membranes are moderately reddened. The appetite is diminished, but the animal chews constantly. Deglutition either of food or water is frequently the cause of spasms of coughing, and these in turn seem to warn the animal against attempts at swallowing. On percussion no alteration is to be detected.

On auscultation mucous rales are heard, with at times tubular breathing ; the latter, however, we will study under the complications, as also the friction warning of pleurisy. Throughout the course of the disease we have still one constant and characteristic symptom—nervous irritability. With a temperature of 104° F. to 107° F., the horse still flinches to the touch on the loins ; stands frequently with head up, and is on the alert for the entrance of anyone to the stall. The previously good tempered and quiet horse will turn and bite, will strike with the hind legs, or at the first touch to the side, head or throat, will half rear and back into the corner of the box, or breaking the halter turn backwards out of the stall.

The *course* of the disease is from five to eight days, but the cough may continue for two or three weeks with variable elevation of temperature. As a stable plague the course is from two to three months. The *termination* is by resolution and recovery, or by complications. In resolution the temperature drops, the cough becomes less frequent and less spasmodic in character, the appetite returns and no sign is left of the disease except the fever mark on the hoof.

The complications are *excessive spasm* and *pleurisy*. In the former the cough may be so violent as to convulse the whole animal, the legs are spread and fixed, with the hind ones drawn slightly under the body. The head and neck are extended, with the muscles tense. The cough comes out by rapidly succeeding efforts, or with the first sound the larynx seems to close for a moment, before the rest can follow. In two cases of my own the spasm has been so great that the animal has fallen to the ground. During these accesses the respiration becomes accelerated, and on auscultation of the trachea and lungs, the tubular murmur of pneumonia can be heard. This false murmur, however, disappears at the end of the attack. In the case which fell to the ground the horse would lie for a moment or two absolutely motionless. (In the first I believed that he had broken his neck.) The rapid respiration was then followed by a long inspiration, the animal regained his feet, the respirations became almost normal and the tubular murmur had disappeared. I have seen no fatal termination from this spasm of the pneumo-gastric, but can readily believe that traumatism might prove fatal, or that the spasm might continue long enough to produce asphyxia. The fatal complication is pleurisy. This occurs when the horse has been kept at work after the development of the disease, and is probably in no way specific, but the result of work on an animal with a high temperature. The symptoms are those of an ordinary pleurisy.

*Diagnosis.*—The diagnosis is based upon the elevation of temperature without corresponding acceleration of the pulse and of respiration ; upon the retention of appetite and reflex, with the great irritability of temper, in the presence of a high temperature, and upon the spasmodic cough and ausculatory sounds of bronchitis, with but trifling discharge.

The diagnosis is made from *Œdematous Pneumonia* by the absence of the yellow colorations, the absence of pneumonia, and the less continuous high temperature from *influenza* by the absence of œdema, of ochre coloration and of the typhoid symptoms; from *strangles* by want of enlarge ment of the lymphatics, absence of purulent discharge and abscesses ; from *variola* by the non-appearance of pustules and enlarged lymphatics ; from *simple bronchitis* as the latter is sporadic, and in it great fever is accompanied by profuse discharge ; from *Rheumatic Pleurisy and Pleurodynia* by the history in these of repeated attacks and great temporary pain ; from *Surgical fever* by the absence of cause.

*Prognosis.*—The prognosis is usually favorable. This disease entails only the loss of ten days to three weeks' use of the animal, and leaves the subject with no complicating sequelæ. In some cases I have seen the irritable disposition remain for a length of time, but in every case it has finally disappeared.

As I have suggested, violent spasm might prove fatal. Pleurisy would render the prognosis serious, as the same disease would when occurring from simple causes.

*Treatment.*—The treatment of a stable should be at once prophylactic. The infected animals should be removed, and complete disinfection of the stalls and area should be made. The individual treatment is simple. The hygienic measures of cleanliness, fresh air without draughts, frequent rubbing and tempting food should be thorough. The digestive tract is to be regulated by small doses of Bi-carbonate of Soda, Sulphate of Soda, Gentian and Tannic Acid. The appetite is to be stimulated by drinks of cold breakfast tea and cow's milk. Anti-spasmodics are to be used when the cough is excessive—the best of these are camphor, belladonna, stramonium and steaming with turpentine. [Turpentine one ounce, water half a bucket.] External frictions of alcohol and turpentine, with hot packs to the loins, will also afford relief. Quinine and Salicylic acid may be used during the elevation of temperature. Prof. Dieckerhoff recommends tracheal injections in ounce doses of the following solution: Acetate of Aluminium, one per cent.; Alum, one-half to one per cent.; Bromide of Potash, one per cent. to two per cent.; water, one hundred.

